# The Relationship between Selected Body Composition Components and Cardiopulmonary Resuscitation Parameters in Nurses: An Observational Simulation Study

**DOI:** 10.3390/jcm11010049

**Published:** 2021-12-23

**Authors:** Paweł Więch, Marek Muster, Łukasz Godek, Izabela Sałacińska, Edyta Guty, Grzegorz Kucaba, Dariusz Bazaliński

**Affiliations:** 1 Institute of Health Sciences, College of Medical Sciences, University of Rzeszow, 35-959 Rzeszów, Poland; mmuster@ur.edu.pl (M.M.); isalacinska@ur.edu.pl (I.S.); dbazalinski@ur.edu.pl (D.B.); 2 Institute of Social Sciences and Health Protection, East European State Higher School in Przemyśl, 37-700 Przemyśl, Poland; e.guty@pwsw.eu; 3 Institute of Physical Culture Studies, College of Medical Sciences, University of Rzeszow, 35-959 Rzeszów, Poland; lgodek@ur.edu.pl; 4 Institute of Medical Sciences, College of Medical Sciences, University of Rzeszow, 35-959 Rzeszów, Poland; gkucaba@ur.edu.pl

**Keywords:** body composition, resuscitation, nutritional indicators, chest compression, ventilation

## Abstract

The provision of cardiopulmonary resuscitation (CPR) may be related to the physical parameters of the medical personnel, including fat mass (FM) and fat-free mass (FFM) components. In this study, we aimed to assess the relationship between selected body composition components and chest compression and ventilation parameters provided by medical staff. An observational simulation study was undertaken between December 2017 and January 2019 at the Center for Innovative Research in Medical and Natural Sciences of Rzeszów. In all participants (505 nurses, 37.71 y ± 12.16), the body weight and height were measured and the body mass index (BMI) was calculated. The body composition indicators were obtained using a bioelectrical impedance device, AKERN BIA 101. Afterwards, all participants performed CPR sequences (30 chest compressions and rescue for 2 breaths) for 2 min on a Laerdal Resusci Anne simulator placed on an examination couch with a self-inflating bag and a face mask. Our observations proved that high values of the anthropometric, nutritional and body composition parameters of the medical staff demonstrated a positive significant correlation with the depth and rate chest parameters and were inversely related to the chest adequate recoil. No statistically significant differences were found between the FM or FFM components and ventilation parameters. This study showed that nutritional status and body composition components may be important factors affecting the quality of CPR.

## 1. Introduction

According to the European Resuscitation Council Guidelines 2021 [1], a sudden cardiac arrest (SCA) is the third major cause of mortality in Europe. In the United States, an in-hospital cardiac arrest (IHCA) develops in over 290,000 adults every year and the presenting rhythm on defibrillators and monitors is usually (81%) non-shockable (asystole or pulseless electrical activity (PEA)) [2]. Factors affecting survival after an IHCA are recognized; however, the incidence and outcome are subject to a significant variation [1]. Currently, high-quality cardiopulmonary resuscitation (CPR) is known to be a crucial element of “the Chain of Survival” [3]. Numerous studies have proven a considerable relationship of survival outcomes and CPR quality represented by the thorax compression [4,5,6,7] and ventilation [8] parameters.

The delivery of effective CPR is challenging [9], especially with regard to high-intensity physical exercise decreasing considerably as time passes [10]. Several physiological constraints may seriously compromise the CPR result [9,11]. Performing CPR for certain periods of time is affected by numerous physiological parameters such as muscle fatigue, the heart rate or pulmonary ventilation, which can make a difference to the final effect [10]. In the hospital environment, medical staff need to conduct CPR whilst standing; they must then position their hands on the chest of the patient and maintain straight elbows, which is extremely exhausting [12]. As muscle mass and back muscle strength are essential in physical activities and maintaining posture, their impact on the quality of chest compressions were also the subject of an analysis in a student group [13]. Moreover, based on current scientific research, the quality of CPR depends on other physiological parameters such as height [14], body weight [15,16], body mass index [14,17] or body fat [18]. Therefore, we aimed to evaluate the relationship between selected body composition components and chest compression and ventilation parameters provided by nurses in our study.

To the best of our knowledge, this is an original study that was conducted among a large medical group, focusing solely on the relationship of the anthropometric measurements, nutritional indicators and deep body composition components with the compression and ventilation parameters. Several other articles [12,13,18] assessing body composition parameters on smaller groups and other BIA measuring tools are available.

## 2. Materials and Methods

### 2.1. Ethics

Prior to the study, the research protocol was approved by the institutional Bioethics Committee at the University of Rzeszów (Resolution No. 11/10/2016) and by all appropriate administrative bodies. The study followed the ethical standards included in an appropriate version of the Declaration of Helsinki and in Polish national regulations.

### 2.2. Subjects

The present observational study was conducted between December 2017 and January 2019 at the Center for Innovative Research in Medical and Natural Sciences of the University of Rzeszów among women. The study involved a group of 505 nurses (37.71 y ± 12.16) active in their profession and certified to perform in the nursing profession, working in selected hospital wards (hospital emergency department, internal medicine, neurology, cardiology, surgery, anesthesia and intensive care) and attending a full-time Masters program in Nursing. Study subjects were recruited by the scientific society of the students at the College of Medical Sciences, the research and teaching staff of this Faculty and the local social networks of the nurses attending postgraduate Masters studies in Health Sciences. Each participant was asked to answer a number of socio-demographic questions: work experience (12.4 y ± 12.87), specialization in a nursing field (22.2%), qualification course (46.9%) and the CPR course content during the previous 5 years (17.6%). Each nurse participating in the study had a basic knowledge and skills in the studied field. The inclusion criteria included: age over 18, having the right to practice as a nurse, a lack of disease or dysfunction making it impossible for the person to perform full CPR, a lack of acute or chronic disease that may have an effect on their nutritional status and a lack of contraindications specified by the company producing the bioimpedance (BIA) analyzer. Participation in the study was voluntary and anonymous. Nurses who failed to express informed consent or who were enrolled in another study were excluded. The purpose of the study with its protocol was explained. Out of 532 nurses initially invited to participate in the study, 505 nurses were finally included (of the 27 nurses excluded, 23 did not meet the inclusion criteria and 4 declined to participate).

### 2.3. Assessments

The participants performed single rescuer CPR according to the “hospital” protocol (manikin positioned on the examination couch 51 cm above the floor, an open airway, check breathing, start 30 compressions then 2 bag valve mask ventilations) for 2 min using a manikin (Laerdal Resusci Anne^®^, 50 kg, Laerdal, Norway). A Laerdal silicone resuscitator (LSR) for adults > 25 kg, volume 1600 mL, and a silicone mask (adult 4–5 with a multi-functional mask cover, Laerdal, Norway) were applied. The European Resuscitation Council (ERC) guidelines [19] were presented before the study (depth of chest compression 5–6 cm, full relaxation, compression rate 100–120/min, tidal volume 6–7 mL/kg). During the task, no feedback was given. The data concerning compression and relaxation were saved on a SimPad^®^ PLUS (SimPad system operating Ubuntu Linux, version 14.04; Laerdal, Taiwan) and analyzed on a computer by means of dedicated software (Laerdal Resusci Anne^®^ Skill Reporter™, Laerdal, China). The parameters subjected to the analysis included: adequate recoil, adequate depth, adequate rate, average rate of all compressions, average depth, number of compressions, number of ventilations, average tidal volume, minute volume, ventilation with adequate volume and average rate of all ventilations during the session. The CPR parameter characteristics are presented in Table 1.

Before the CPR procedure, the body weight and height of the participants were measured. The body height was measured with a Seca 213 portable stadiometer to the nearest 0.1 cm. During the test, the participants were asked to stand barefoot in an upright position with their back to the stadiometer. The average of three measurements was taken for the analysis. The body weight was assessed with a precision of 0.1 kg by means of a digital scale (Radwag 100/200 OW, Radom, Poland).

Subsequently, the BIA analysis was conducted by an AKERN BIA 101 Anniversary Sport Edition Analyzer (Akern SRL, Pontassieve, Florence, Italy) to investigate the nutritional status presented by selected indicators (body mass index (BMI), basal metabolic rate (BMR), body cell mass index (BCMI) and phase angle (PA)) and body composition parameters (fat mass (FM), fat-free mass (FFM), muscle mass (MM), total body water (TBW) and body cell mass (BCM)). The results were subjected to the analysis by dedicated software (Bodygram1_31 from AKERN, Pontassieve, Florence, Italy).

The BIA output parameters (R, resistance, the opposition offered by the body to the flow of an alternating electrical current, and Xc, the reactance related to the capacitance properties of the cell membrane) were measured between 7:00 and 12:00 in a fasting state and in a supine position with the upper (30°) and the lower (45°) limbs abducted after no less than 5 min of rest. A tetrapolar system with a contralateral mode was applied. The current amplitude was 800 μA and the sinusoidal, 50 kHz. The reliability and repeatability of the results were ensured by subsequently performing two measurements. Disposable electrodes were attached to the dorsal surface of a right upper (over the wrist) and a right lower extremity (on the ankle). All the measurements were taken according to the guidelines described by other authors [20,21,22,23]. The calculations utilized by the software to analyze the specific parameters are the restricted property of the company. The body composition characteristics of the study group are presented in Table 2.

### 2.4. Statistical Analysis

The statistical analysis was conducted with Statistica 13.1 (StatSoft Inc., Kraków, Poland). The Shapiro–Wilk test was used to verify the equivalence of the studied groups and the compliance of the distribution of variables with a normal distribution. A Spearman’s rank correlation test was used because the variables did not conform to the normal distribution. The prevalence was calculated with a 95% confidence interval. A *p*-value below 0.05 was considered to be statistically significant.

## 3. Results

Table 3 presents the essential characteristics of the basic anthropometric (body weight, height) and impedance (resistance, reactance) parameters in the study group. Significant positive correlations were identified between the body weight of the participants and an average (r = 0.39, *p* < 0.001) or adequate (r = 0.40, *p* < 0.001) depth. A higher level of adequate recoil was also negatively associated with the body weight of the participants (r = −0.25, *p* < 0.001). Furthermore, the correlations between the height and average (r = 0.18, *p* < 0.001) or adequate (r = 0.18, *p* < 0.001) depth had a positive tendency.

The studies also showed that all presented indicators were negatively significantly correlated with adequate recoil (PA: r = −0.13, *p* = 0.005; BMR: r = −0.09, *p* = 0.048; BMI: r = −0.23, *p* < 0.001; BCMI: r = −0.21, *p* < 0.001) and positively correlated (apart from PA) with adequate depth (BMR: r = 0.09, *p* = 0.048; BMI: r = 0.33, *p* < 0.001; BCMI: r = 0.21, *p* < 0.001) and average depth (BMR: r = 0.09, *p* = 0.046; BMI: r = 0.33, *p* < 0.001; BCMI: r = 0.21, *p* < 0.001). For BMR only, the correlations with all ventilation parameters were statistically significant (Table 4).

As observed in Table 5, significant differences appeared between the fat mass or fat-free mass and selected compression parameters. Adequate recoil was negatively correlated with FM (kg and %) and FFM (kg) whereas higher levels of average and adequate depth were associated with a significantly increased FM (kg and %) and FFM (kg). Only the percentage of FFM showed inverse correlations with the abovementioned compression parameters. No statistically significant differences were found between the fat or fat-free mass components and ventilation parameters, respectively.

**Figure 1:**
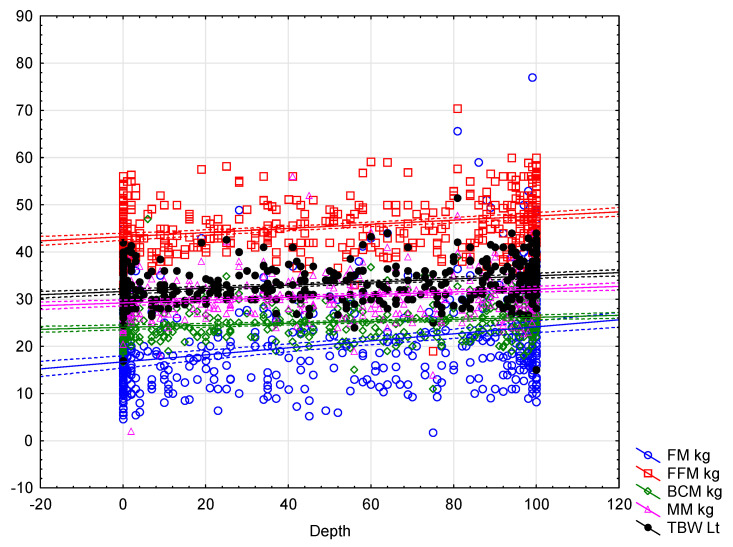
Comparison of adequate depth and body composition parameters.

**Figure 2:**
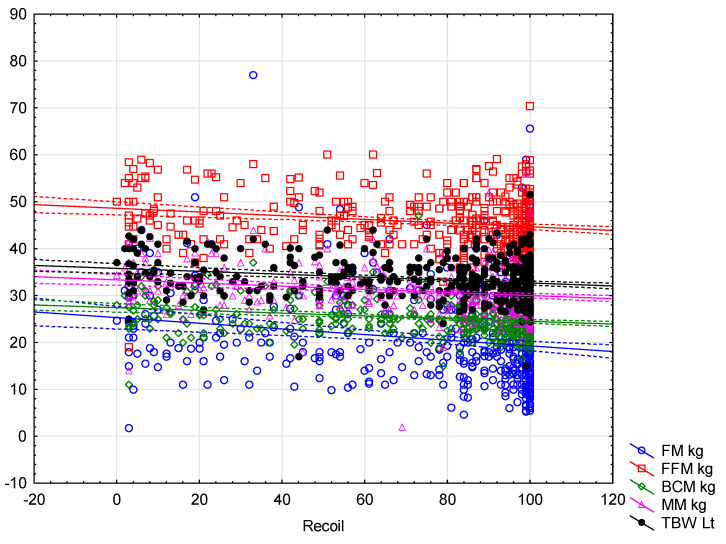
Comparison of adequate recoil and body composition parameters.

Finally, only selected compression parameters were significantly positively or negatively correlated with the fat-free mass components represented by BCM kg (adequate recoil: −0.23, *p* < 0.001; adequate and average depth: 0.28, *p* < 0.001), BCM % (adequate recoil: −0.14, *p* = 0.002), MM kg (adequate recoil: −0.23, *p* < 0.001; adequate depth: 0.30, *p* < 0.001; average depth: 0.29, *p* < 0.001), MM % (adequate recoil: 0.12, *p* = 0.007; adequate depth: −0.27, *p* < 0.001; average depth: −0.26, *p* < 0.001), TBW Lt (adequate recoil: −0.21, *p* < 0.001; adequate and average depth: 0.36, *p* < 0.001) and TBW % (adequate recoil: 0.20, *p* < 0.001; adequate depth: −0.28, *p* < 0.001 and average depth: −0.27, *p* < 0.001) (Table 6). A linear regression analysis between depth and the recoil vs. selected BIA parameters are presented in Figure 1 and Figure 2.

## 4. Discussion

This study presented data regarding the comparison of anthropometric and body composition components with chest compression and ventilation parameters in nurses as well as their potential impact on in-hospital CPR quality. Interestingly, significant correlations were identified in most compression parameters but few ventilation ones in the examined group of medical personnel. When comparing our own results with the research of other authors, it should be remembered that our study group consisted of women only, which is related to the feminization of the nursing profession performed by generations both in Poland and in the world. Therefore, the results obtained by us should be considered in the context of in-hospital CPR by nurses as the first witnesses of an IHCA and the quality of the performed activities. Gender may influence the coexistence of differences in the number, percentage and distribution of body composition components, which may translate into the quality of compression and ventilation parameters. Large epidemiological studies assessing the body weight of the Italian adult population showed higher percentages of body fat in women (33.6%) vs. men (22.1%) in each of the studied age groups [24]. The above observations were also confirmed in other population studies of adults [25,26] with a particular emphasis on the percentage of higher mean median FM in women [27,28,29,30].

Generally, a greater body weight should lead to better chest compression results. Our research confirmed that theory because significant positive correlations were found between the body weight of the participants and average (r = 0.39, *p* < 0.001) or adequate (r = 0.40, *p* < 0.001) depth. Moreover, the correlations between height and BMI vs. the depth of the chest compression had a positive tendency. Our results are consistent with the study of Gianotto-Oliveira et al., which found a greater depth of chest compressions in the overweight participants [31]. Reddy at al. also observed an adequate depth of chest compressions correlated positively with BMI [32]. Findings presented by López-González et al. confirmed that underweight participants obtained lower results than the normal weight and overweight/obese participants in variables such as correct compression depth (*p* < 0.001) and adequate external chest compressions (*p* < 0.001) [33]. During an in-hospital resuscitation, medical staff generally need to perform CPR whilst standing with their hands on the chest of the patient. A greater rescuer height makes it easier to obtain an angle of 90 degrees between the upper limbs and the chest of the patient. However, a greater body weight influencing the depth of the chest compression does not necessarily determine the effectiveness of the resuscitation. Chest recoil may also play a crucial role. In our observation, a higher level of adequate recoil was negatively associated with the body weight (r = −0.25, *p* < 0.001), BMI (r = −0.23, *p* < 0.001), BMR (r = −0.09, *p* = 0.048) and PA (r = −0.13, *p* = 0.005) of the participants. This suggested that the participants with a higher body weight and higher basic caloric requirement were more effective at obtaining a better depth but at the expense of permitting the recoil of the chest. Based on our clinical observations, we concluded that an improvement in the depth of the chest compression had an impact on the other compression parameters described in the ERC Guidelines 2021 [19]. However, it should be emphasized that weight or a BMI analysis may indicate unreliable results in a few circumstances. The index does not capture constant changes to the body composition, muscle development, skeletal resorption and increase in cell mass due to it relying solely on anthropometric measurements.

Previous studies [12,13,18] have found that body composition components are essential to understand the mechanism of resuscitation efforts. Our study exclusively focused on analyzing the differences between fat mass and specific fat-free mass components such as body water, muscle mass or body cell mass in relation to CPR parameters. In this study, an increased FM and FFM were significantly positively associated with higher levels of average and adequate depth. Finally, the FFM components were significantly positively correlated with selected compression parameters (BCM kg vs. adequate and average depth: r = 0.28, *p* < 0.001; MM kg vs. adequate depth: r = 0.30, *p* < 0.001; MM kg vs. average depth: r = 0.29, *p* < 0.001 and TBW Lt vs. adequate and average depth: r = 0.36, *p* < 0.001). Despite limited research in this area, a few partially confirm our observations. The study by Kaminska et al. revealed that BMR and FFM as well as trunk muscle mass correlated positively with the chest compression depth (*p* < 0.05). No significant correlations between FM% and chest compression depth or rate were observed [18]. In a study of 70 active firefighters, a significant positive correlation between the upper extremity muscle mass with the core muscle mass and chest compression depth was noted (r = 0.398, *p* = 0.001). A lack of significant correlations between the CPR quality and body fat percentage was also observed (*p* > 0.05) [12]. It is worth mentioning that the results of the pilot study by Shin et al. showed that FM% was significantly negatively correlated with the compression depth (r = −0.745, *p* = 0.001) and coexisted with non-significance for the skeletal muscles [13]. In our opinion, the difference in the FM correlation trend may be due to differences in the size of the group (*n* = 505 in own study vs. *n* = 16 in the pilot study).

The interpretation of the results should also include the recoil parameters. Thus, adequate recoil correlated negatively with FM (r = −0.22, *p* < 0.001) and FFM, represented by BCM kg (r = −0.23, *p* < 0.001), MM kg (r = −0.23, *p* < 0.001) and TBW Lt (r = −0.21, *p* < 0.001). Our observations clearly showed that people with a higher body mass—both fat and lean—achieved a better depth of compression of the chest and a poorer relaxation. To our knowledge, this is the first study to analyze the described parameters with reference to body composition components; therefore, a comparison with the results of other studies is impossible. Additionally, in our observations, a lack of statistically significant differences was found between the above components and all ventilation parameters. Despite the lack of outcome data presented by other authors, the analysis of the correctness of the ventilation parameters in relation to various factors (including body composition components) should be continued in future studies.

The research presented by us may indicate the direction of further work of nurses and other medical workers on the technique of performing CPR in real or simulated conditions. Rescuers with a large body weight reflected in the FM and FFM components coping well with pressure should also be more focused on increasing the percentage of chest relaxation in order to increase the effectiveness of the actions over time. Medical workers with lower parameters of FM and FFM performing a correct chest relaxation should strive to optimize their resuscitation position (its stability; maintaining a straight line between the arms, elbows and wrists; obtaining a 90 degree angle in relation to the chest), which allows for a lower energy expenditure per unit of time and optimizes the compression parameters. In addition, we believe that the initial anthropometric and body composition parameters of the rescuer should be taken into account in external and internal CPR training for healthcare professionals to optimize the operational efficiency.

The limitations of this study may have had an impact on the results. First of all, all subjects were women (*n* = 505) of 37.71 y ± 12.16, which could slightly complicate the comparison with other studies. Despite the homogeneity of our group, in our opinion the general result trend did not change. Moreover, the large size and homogeneity of the group allowed for a lower risk of an estimation error and a more reliable inference. On one hand, we drew attention to the strength of the correlation determined by the Pearson (R) test; general assumptions were made in this matter (i.e., the low strength of the relationship with absolute values < 0.3) and the value in many outcome parameters was low. On the other hand, we assumed that with a large group size, the strength of the relationship would be low but the statistical significance high. Our goal in the study, apart from the analysis of these relationships, was also to indicate the direction of these changes, which may be of great importance to people performing resuscitation in the context of its quality and the factors influencing it. Secondly, it should be also mentioned that the manikin did not accurately reflect the conditions of a real patient; however, it is an important and proven tool for teaching as well as improving CPR knowledge and skills. It should be emphasized that new data correlations related to depth, frequency and the relaxation of CPR can realistically affect the survival of patients. Moreover, new data and interesting findings can be found in our study, which may be indicative for the directions of future research.

## 5. Conclusions

These results strongly indicated that nutritional status and body composition components may be significant factors affecting the quality of CPR. High values of anthropometric (weight, high), nutritional (BMI, PA, BCMI) and body composition (FM, FFM, BCM, MM, TBW) parameters of medical staff predicted better compression results. The relationship was inversely related to an adequate chest recoil for all the parameters listed. Weight and body composition did not significantly affect the efficiency of chest ventilation.

## Figures and Tables

**Table 1:** Chest compression and ventilation parameters of the study participants.

Variables	Descriptive Statistics		
	Median	Q1	Q3
Adequate recoil (%)	95.00	67.00	100.00
Adequate depth (mm)	55.00	3.00	96.00
Adequate rate (x/min)	20.00	1.00	73.00
Average rate of all compressions (*n*)	116.00	101.00	129.00
Average depth (mm)	50.00	43.00	56.00
Number of compressions (*n*)	150.00	135.00	169.00
Number of ventilations (*n*)	3.00	0.00	7.00
Average tidal volume (mL)	267.00	0.00	456.00
Minute volume (mL/min)	896.00	0.00	3216.00
Ventilation with adequate volume (*n*)	0.00	0.00	40.00
Average rate of all ventilations (*n*)	1.00	0.00	3.00

**Table 2:** Body composition parameters of the study participants.

Variables	Descriptive Statistics			
	Mean	Min	Max	SD
Fat mass (kg)	20.54	1.70	77.00	9.71
Fat mass (%)	30.11	8.00	315.00	14.90
Fat-free mass (kg)	45.49	19.00	70.40	5.67
Fat-free mass (%)	70.43	35.00	92.00	7.80
Body cell mass (kg)	25.13	11.00	47.00	3.73
Body cell mass (%)	54.79	44.00	67.50	3.05
Muscle mass (kg)	30.71	2.00	56.00	4.68
Muscle mass (%)	47.45	32.00	67.30	5.82
Total body water (Lt)	33.33	15.00	51.50	4.17
Total body water (%)	51.70	4.71	123.00	6.62
Phase angle (°)	6.26	4.30	9.50	0.66
Basal metabolic rate (kcal)	2434.09	1070.00	7869.20	1939.33
Body mass index (kg/m^2^)	24.87	8.90	57.50	5.02
Body cell mass index (kg/m^2^)	10.00	4.60	115.00	7.05

**Table 3:** Comparison of the compression and ventilation parameters with weight, height, RZ and XC.

Chest Compression and Ventilation Parameters	Weight (kg)		Height (cm)		RZ (Ω)		XC (Ω)	
	R	*p*	R	*p*	R	*p*	R	*p*
Adequate recoil	−0.25	**<0.001**	−0.07	0.095	0.19	**<0.001**	0.10	**0.021**
Adequate depth	0.40	**<0.001**	0.18	**<0.001**	−0.28	**<0.001**	−0.26	**<0.001**
Adequate rate	−0.09	0.050	0.02	0.710	0.10	0.020	0.06	0.193
Average rate of all compressions	−0.05	0.282	−0.01	0.865	0.05	0.241	0.06	0.197
Average depth	0.39	**<0.001**	0.18	**<0.001**	−0.29	**<0.001**	−0.27	**<0.001**
Number of compressions	−0.01	0.892	−0.01	0.781	0.03	0.554	0.03	0.513
Number of ventilations	0.06	0.199	−0.02	0.608	−0.04	0.432	−0.08	0.071
Average tidal volume	0.07	0.139	−0.01	0.802	−0.09	**0.045**	−0.11	**0.014**
Minute volume	0.07	0.133	−0.03	0.572	−0.06	0.145	−0.10	**0.022**
Ventilation with adequate volume	0.05	0.311	0.00	0.968	−0.04	0.392	−0.11	**0.010**
Average rate of all ventilations	0.06	0.176	−0.02	0.695	−0.03	0.480	−0.08	0.073

RZ: resistance; XC: reactance. Significant differences are in bold.

**Table 4:** Comparison of the compression and ventilation parameters with nutritional indicators.

Chest Compression and Ventilation Parameters	PA (°)		BMR (kcal)		BMI (kg/m^2^)		BCMI (kg/m^2^)	
	R	*p*	R	*p*	R	*p*	R	*p*
Adequate recoil	−0.13	**0.005**	−0.09	**0.048**	−0.23	**<0.001**	−0.21	**<0.001**
Adequate depth	−0.03	0.494	0.09	**0.048**	0.33	**<0.001**	0.21	**<0.001**
Adequate rate	−0.01	0.830	0.01	0.738	−0.08	0.067	−0.08	0.058
Average rate of all compressions	0.03	0.558	−0.04	0.326	−0.04	0.419	−0.02	0.654
Average depth	−0.03	0.462	0.09	**0.046**	0.33	**<0.001**	0.21	**<0.001**
Number of compressions	0.02	0.671	0.03	0.525	0.02	0.706	−0.00	0.956
Number of ventilations	−0.05	0.221	−0.13	**0.003**	0.07	0.094	0.03	0.480
Average tidal volume	−0.02	0.641	−0.14	**0.002**	0.07	0.128	0.07	0.144
Minute volume	−0.04	0.347	−0.14	**0.001**	0.08	0.069	0.05	0.241
Ventilation with adequate volume	−0.10	**0.018**	−0.23	**<0.001**	0.04	0.399	−0.01	0.817
Average rate of all ventilations	−0.06	0.207	−0.14	**0.001**	0.07	0.104	0.03	0.496

PA: phase angle; BMR: basal metabolic rate; BMI: body mass index; BCMI: body cell mass index. Significant differences are in bold.

**Table 5:** Comparison of the compression and ventilation parameters with fat and fat-free mass components.

Chest Compression and Ventilation Parameters	FM (kg)		FM (%)		FFM (kg)		FFM (%)	
	R	*p*	R	*p*	R	*p*	R	*p*
Adequate recoil	−0.22	**<0.001**	−0.19	**<0.001**	−0.21	**<0.001**	0.19	**<0.001**
Adequate depth	0.34	**<0.001**	0.29	**<0.001**	0.36	**<0.001**	−0.29	**<0.001**
Adequate rate	−0.07	0.128	−0.04	0.407	−0.06	0.160	0.04	0.340
Average rate of all compressions	−0.05	0.290	−0.03	0.552	−0.04	0.319	0.02	0.643
Average depth	0.33	**<0.001**	0.28	**<0.001**	0.36	**<0.001**	−0.28	**<0.001**
Number of compressions	0.01	0.887	0.02	0.615	−0.02	0.736	−0.03	0.526
Number of ventilations	0.05	0.269	0.07	0.142	0.04	0.346	−0.06	0.184
Average tidal volume	0.05	0.276	0.03	0.510	0.08	0.071	−0.02	0.577
Minute volume	0.05	0.252	0.05	0.224	0.06	0.166	−0.05	0.273
Ventilation with adequate volume	0.02	0.670	0.03	0.566	0.04	0.380	−0.03	0.572
Average rate of all ventilations	0.05	0.256	0.07	0.139	0.04	0.323	−0.06	0.181

FM: fat mass; FFM: fat-free mass. Significant differences are in bold.

**Table 6:** Comparison of the compression and ventilation parameters with selected fat-free mass components.

Chest Compression and Ventilation Parameters	BCM (kg)		BCM (%)		MM (kg)		MM (%)		TBW (Lt)		TBW (%)	
	R	*p*	R	*p*	R	*p*	R	*p*	R	*p*	R	*p*
Adequate recoil	−0.23	**<0.001**	−0.14	**0.002**	−0.23	**<0.001**	0.12	**0.007**	−0.21	**<0.001**	0.20	**<0.001**
Adequate depth	0.28	**<0.001**	−0.03	0.565	0.30	**<0.001**	−0.27	**<0.001**	0.36	**<0.001**	−0.28	**<0.001**
Adequate rate	−0.07	0.120	−0.00	0.994	−0.09	0.055	0.04	0.420	−0.08	0.078	0.04	0.425
Average rate of all compressions	−0.03	0.503	0.05	0.280	−0.02	0.733	0.04	0.398	−0.06	0.177	0.03	0.552
Average depth	0.28	**<0.001**	−0.03	0.556	0.29	**<0.001**	−0.26	**<0.001**	0.36	**<0.001**	−0.27	**<0.001**
Number of compressions	0.00	0.993	0.04	0.356	0.02	0.713	−0.00	0.992	−0.03	0.461	−0.02	0.658
Number of ventilations	0.01	0.742	−0.05	0.309	0.02	0.625	−0.07	0.095	0.04	0.417	−0.05	0.254
Average tidal volume	0.05	0.222	−0.01	0.886	0.07	0.141	−0.04	0.405	0.08	0.072	−0.02	0.724
Minute volume	0.03	0.443	−0.03	0.504	0.04	0.334	−0.06	0.149	0.06	0.181	−0.04	0.389
Ventilation with adequate volume	−0.02	0.723	−0.08	0.075	0.01	0.862	−0.07	0.123	0.04	0.337	−0.03	0.533
Average rate of all ventilations	0.01	0.781	−0.05	0.266	0.02	0.608	−0.08	0.085	0.04	0.431	−0.05	0.246

BCM: body cell mass; MM: muscle mass; TBW: total body water. Significant differences are in bold.

## Data Availability

The data presented in this study are available on reasonable request from the corresponding author: pwiech@ur.edu.pl.
